# Microbially Induced Mineralization of Layered Mn Oxides Electroactive in Li Batteries

**DOI:** 10.3389/fmicb.2020.02031

**Published:** 2020-09-10

**Authors:** Laura Galezowski, Nadir Recham, Dominique Larcher, Jennyfer Miot, Fériel Skouri-Panet, François Guyot

**Affiliations:** ^1^Institut de Minéralogie, Physique des Matériaux et Cosmochimie, Sorbonne Université, Muséum National d’Histoire Naturelle, CNRS UMR 7590, IRD 206, Paris, France; ^2^Laboratoire de Réactivité et Chimie des Solides, CNRS UMR 7314, Université de Picardie Jules Verne, Amiens, France; ^3^Réseau sur le Stockage Electrochimique de l’Energie (RS2E), FR CNRS 3459, Amiens, France; ^4^Institut Universitaire de France (IUF), Paris, France

**Keywords:** Mn-oxidizing bacteria, biomineralization, manganese oxide, electroactivity, electrode materials, Li-ion battery

## Abstract

Nanoparticles produced by bacteria, fungi, or plants generally have physicochemical properties such as size, shape, crystalline structure, magnetic properties, and stability which are difficult to obtain by chemical synthesis. For instance, Mn(II)-oxidizing organisms promote the biomineralization of manganese oxides with specific textures under ambient conditions. Controlling their crystallinity and texture may offer environmentally relevant routes of Mn oxide synthesis with potential technological applications, e.g., for energy storage. However, whereas the electrochemical activity of synthetic (abiotic) Mn oxides has been extensively studied, the electroactivity of Mn biominerals has been seldom investigated yet. Here we evaluated the electroactivity of biologically induced biominerals produced by the Mn(II)-oxidizer bacteria *Pseudomonas putida* strain MnB1. For this purpose, we explored the mechanisms of Mn biomineralization, including the kinetics of Mn(II) oxidation, under different conditions. Manganese speciation, biomineral structure, and texture as well as organic matter content were determined by a combination of X-ray diffraction, electron and X-ray microscopies, and thermogravimetric analyses coupled to mass spectrometry. Our results evidence the formation of an organic–inorganic composite material and a competition between the enzymatic (biotic) oxidation of Mn(II) to Mn(IV) yielding MnO_2_ birnessite and the abiotic formation of Mn(III), of which the ratio depends on oxygenation levels and activity of the bacteria. We reveal that a subtle control over the conditions of the microbial environment orients the birnessite to Mn(III)-phases ratio and the porosity of the assembly, which both strongly impact the bulk electroactivity of the composite biomineral. The electrochemical properties were tested in lithium battery configuration and exhibit very appealing performances (voltage, capacity, reversibility, and power capability), thanks to the specific texture resulting from the microbially driven synthesis route. Given that such electroactive Mn biominerals are widespread in the environment, our study opens an alternative route for the synthesis of performing electrode materials under environment-friendly conditions.

## Introduction

Biologically induced mineralization of manganese is widespread in the environment (Tebo et al., 2004), and results from Mn(II) oxidation to Mn(III) and/or Mn(IV), either promoted by reaction with reactive oxygen species or mediated, in the presence of dioxygen, by the activity of a microbial multicopper oxidase (Learman et al., 2011; Geszvain et al., 2013). Multicopper oxidase-mediated oxidation is coupled to a four-electron reduction of dioxygen into water (Granja-Travez and Bugg, 2018). Because the oxidation of Mn(II) is very slow abiotically, Mn(II)-oxidizing microorganisms play a key role for accomplishing this reaction in terrestrial and aquatic environments (Tebo et al., 2005; Yang et al., 2013). Mn oxides produced by biological activity are generally characterized by a nanometric size and a poorly crystallized structure, which both depend on the microorganism and the culture conditions. For instance, the final product formed by the Mn-oxidizing bacteria *Bacillus* sp. SG-1 is either a hydrated layered phyllomanganate (buserite) or feitknechtite (β-MnOOH), depending on the Mn(II) concentrations (Bargar et al., 2005; Webb et al., 2005). Biominerals formed by the fungus *Paraconiothyrium* sp. WL-2 (Yu et al., 2013) exhibit a phyllomanganate structure, while *Leptothrix discophora* SP-6 produces a todorokite-like structure (Kim et al., 2003). Finally, the strain *Pseudomonas putida* MnB1 produces birnessite with a hexagonal layer symmetry (Villalobos et al., 2003, 2006; Lanson et al., 2004; Toner et al., 2006).

Some Mn^4+^-based oxides (e.g., γ-MnO_2_, ramsdellite/nsutite) have the ability to host cations (e.g., H^+^, alkali metal cations) within their structures, depending on the redox conditions. This property accounts for these Mn oxides to be electrochemically active; hence their widespread use in electrochemical cells such as primary Zn/MnO_2_ cells (Leclanché, alkaline cells, 1.5 V) or Li/MnO_2_ coin-cells (3 V). Among phyllomanganates, birnessite consisting in stacked layers of edge-sharing MnO_6_ octahedra provides 7-Å-wide interplanar spaces wherein cations can reversibly insert. This property accounts for phyllomanganate Mn oxides to be electrochemically interesting for battery energy storage, either vs. lithium, sodium, or magnesium, hence usable in solid-state batteries by providing high capacities of about 300 mAh g^–1^ (Amade et al., 2011; Thapa et al., 2014; Alfaruqi et al., 2015; Wang et al., 2018), with a good potential of 3.2 V vs. Li^+^/Li°.

Unfortunately, the reversibility of these reactions is still an issue precluding the commercialization of efficient rechargeable aqueous MnO_2_-based secondary batteries. The texture and the morphology of these oxides are highly sensitive to environmental parameters (e.g., Marafatto et al., 2017). As a consequence, multiple abiotic synthesis routes have been proposed for various applications, e.g., for their use as electrode materials in supercapacitors requiring powders with high active surface area (Lee and Goodenough, 1999; Jeong and Manthiram, 2002; Toupin et al., 2002; Kim and Popov, 2003; Brousse et al., 2006; Alfaruqi et al., 2015; Qiu et al., 2018). At laboratory or industrial scales, the precipitation of MnO_2_ from Mn-bearing solutions is generally triggered by the use of strongly oxidizing conditions, obtained either chemically (e.g., peroxodisulfate Na_2_S_2_O_8_) or electrochemically (e.g., by applying an oxidizing current), both under acidic conditions, i.e., under non-environment-friendly conditions. In addition, pH modifications can affect the texture of the Mn oxides formed through a quantitative tuning of the precipitation rates (Zhao et al., 2016). However, some structural and/or textural aspects are still out of neat control.

With the aim of bypassing this technological barrier, biomineralization, bio-assisted, or bio-inspired processes can be used as eco-efficient methods for fabricating Li-ion electrodes. Microbial biomineralization proceeding under soft conditions, i.e., at ambient temperature and in aqueous media, provides nanoparticles with specific properties (crystallinity, texture, catalytic activity, and electroactivity) usually difficult to reach through abiotic pathways (Kim et al., 2018; Piacenza et al., 2018; Capeness et al., 2019; Gallardo-Benavente et al., 2019; Gomez-Bolivar et al., 2019). This has been reported for the microbial-assisted Li-ion electrode synthesis of Fe phosphates (Mirvaux et al., 2016), Fe-oxides (Miot et al., 2014), Co_3_O_4_ (Shim et al., 2011; Rosant et al., 2012), MnO*_*x*_*/C (Li et al., 2016), or MnO_2_ (Yu et al., 2013). In all these studies, the benefits of microbial-induced mineralization arose from the specific texture of the biominerals stemming from their microbial origin.

In the present study, our goal was to produce manganese oxides as active material for Li battery through bacterial synthesis under environment-friendly conditions. We performed experiments with *P*. *putida* strain MnB1 that use a protein homologous to multi-copper oxidases to induce Mn oxidation and mineralization. We evaluated the electrochemical reactivity vs. lithium of the biomineralized Mn-bearing products. We provide new data on the kinetics of Mn(II) oxidation by *P*. *putida* and on the nature of the Mn-bearing biominerals formed. In addition, we evaluated the impact of culture oxygenation on the kinetics of Mn(II) oxidation and the influence of Mn(II) addition on the nature of the Mn oxides formed, both under abiotic and biotic conditions. Manganese speciation, mineral crystallinity, and texture as well as organic matter content were determined by a combination of X-ray diffraction, scanning transmission x-ray microscopy (STXM), electron microscopies [scanning electron microscopy (SEM) and transmission electron microscopy (TEM)], and thermogravimetric analyses coupled to mass spectrometry. We evidence the enhanced electrochemical performance of the mineral–organic matter composite obtained by biomineralization compared to abiotic counterparts, which opens an alternative route for electrode material synthesis under environment-friendly conditions.

## Materials and Methods

### Bacterial Culture and Production of Biomineralized Mn Oxides

All solutions for culture media were sterilized by autoclaving. The manganese oxides were produced in cultures of *P*. *putida* strain MnB1 (ATCC^®^ 23483^TM^). All cultures were performed at 30°C, under aerobic conditions using an incubator system, with horizontal stirring (120 rpm). First, the bacteria were pre-cultured overnight in growth medium, pH 6.8, composed of beef extract (3 g L^–1^), peptone (5 g L^–1^), and MnSO_4_⋅H_2_O (50 μM), after inoculation at 1/60 (v/v) from a stock culture stored at 4°C. Then, the cells were rinsed three times in HEPES 10 mM + NaCl 10 mM, then transferred in stationary phase into 150 ml (Mn-Bio_150__ml_) or 1 L (Mn-Bio_1__l_) of mineralization medium at a cell density of 2.3 10^8^ cells/ml. The mineralization medium (pH 7.40) was composed of HEPES (10 mM), (NH_4_)_2_SO_4_ (2 mM), NaCl (0.7 mM), and glucose (1 mM). MnSO_4_⋅H_2_O (0.2 mM) was added daily (1.5 ml of MnSO_4_⋅H_2_O 10 mM). The mineralization experiments were performed over 2 weeks. Two independent Mn-Bio_150__ml_ experiments comprising five replicates were performed exactly under the same conditions and used to follow Mn concentrations. The first experiment (performed in duplicate) was used to provide all the other results reported in the main text of this paper. The results obtained for the second experiment (three replicates) are provided in the supporting information (Supplementary Figures SI3, SI5). Abiotic controls were prepared in the same way but without bacterial inoculation.

### Abiotic Reference Compounds

Mn-bearing minerals were used as reference compounds for STXM analyses. Manganese oxide reference and HMnPO_4_⋅3H_2_O were synthetized in solution. HMnPO_4_⋅3H_2_O was synthetized by solubilizing 1.275 g MnSO_4_ and 1 g KH_2_PO_4_ in 15 ml of water; then, the solution was transferred in an autoclave (Parr^®^ 23 ml). Thereafter, the solution was heated at 160°C overnight. Manganese oxide was synthetized by a modified procedure from Villalobos et al. (2003), i.e., the following reagent solutions were prepared:

• 1 g KMnO_4_ in 100 ml MQ water [solution (1)].
• 2.4 g MnCl_2_⋅4H_2_O in100 ml MQ water [solution (2)].
• 0.7 g NaOH in 100 ml MQ water [solution (3)].

Solution (1) was added slowly to solution (3) under stirring. Solution (2) was then added to the previous mixture and maintained under stirring overnight. The resulting precipitate was washed three times in water. The precipitate was then incubated in 150 ml of 1 mol/L NaCl under stirring for 1 h. All powders were then washed twice in water and once in acetone and finally dried at 80°C in air. The Mn(III)-to-Mn(IV) ratio of abiotic compounds was estimated by redox titration in Mohr’s salt using permanganometry (Elias, 2002).

### Monitoring of Mn Aqueous Concentrations

**FIGURE 1:**
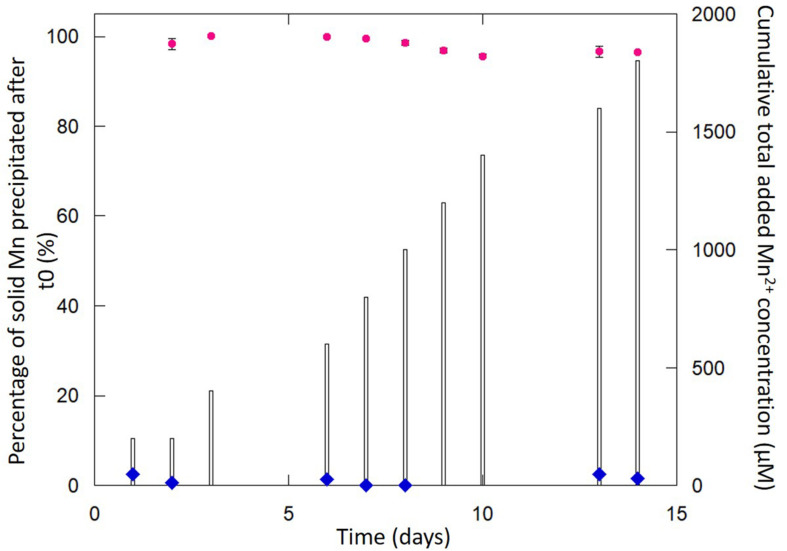
Evolution of the percentage of solid Mn precipitated in Mn-Bio_150__ml_ (pink circles correspond to the mean of five replicates in two independent cultures and error bars correspond to the standard deviation). Cumulative total added Mn^2+^ concentration (bars) increases with time following daily additions of Mn^2+^. The abiotic control (blue diamonds) does not show any Mn precipitation even after 14 days.

The chemical measurements obtained for the two independent Mn-Bio_150__ml_ experiments comprising five replicates are reported in Figure 1. Dissolved manganese concentrations were measured daily by inductively coupled plasma atomic emission spectroscopy (ICP-AES, Cetac ASX-520) after acidification in 2% HNO_3_ (Suprapur, Sigma Aldrich) to keep the analytes of interest in the solution prior to being nebulized, followed by 0.22-μm filtration.

### Mn(II) Addition Experiments

All these experiments were performed in duplicate, at 30°C, under aerobic conditions, with horizontal stirring (120 rpm) and without oxidizing bacteria inoculation. Then, 30 mg of biogenic birnessite obtained from 150 ml of *P*. *putida* cultures (Mn-Bio_150__ml_) was dried and added in 50 ml of mineralization medium. Drying of the powder at 80°C inactivated the bacterial cells. MnSO_4_.H_2_O was added daily (up to 1.8 mM). Dissolved manganese concentrations were measured daily by ICP-AES (Cetac ASX-520) after acidification in 2% HNO_3_ (Suprapur, Sigma Aldrich) followed by 0.22-μm filtration.

### Analyses of Solids

Solids were collected from biomineralization and abiotic experiments by centrifugation (4,000 *g*, 10 min) and washed three times in water. The samples were further dried at 80°C overnight. The proportions of mineral and organic matter in the materials produced in the presence of *P*. *putida* were quantified by thermogravimetric analysis (TGA), using a STA449C coupled to a quadrupole mass spectrometer (QMS 403 Aeolos), under dry air flow (50 ml min^–1^) with a ramp of 5° min^–1^ up to 900°C. These analyses were coupled with differential scanning calorimetry (DSC). The crystalline minerals were identified by X-ray diffraction (XRD) performed in capillaries with a Rigaku MM007HF X-ray diffractometer using a rotating molybdenum anode and a RAXIS4++ imaging plate detector. Spatially resolved Mn speciation was determined by X-ray absorption spectroscopy using STXM at the Mn L_2,3_-edges (630–660 eV). The measurements were performed at the HERMES beamline (SOLEIL, Saint-Aubin, France) (Belkhou et al., 2015) under vacuum. Image stacks were acquired following previously published procedures (Miot et al., 2014). Beam damage was quantified by monitoring the spectral changes at the Mn L_2,3_-edges at increasing dwell times of up to several tens of milliseconds. All other measurements were performed with a dwell time between 1 and 3 ms. The Mn L_2,3_-edges near-edge X-ray absorption fine structure spectra were extracted from image stacks and normalized following the procedure of Bourdelle et al. (2013) adapted to the Mn L_2,3_-edges, i.e., in a first step, a linear background correction was applied between 631 and 638 eV, then in a second step, w1 and w2 were fixed to 1 eV, *E1* between 640 and 642 eV, and *E2* between 650 and 652 eV. Here w1 and w2 are L_3_ and L_2_ peak widths, respectively, and E1 and E2 are the energies of the inflection points near the L_3_ and L_2_ edge onsets, respectively. Normalized curves were fit as a linear combination of reference compound spectra. Data were processed using the aXis2000 software (Hitchcock, 2001). Mn speciation maps were calculated from the image stacks by singular value decomposition using the stack–fit routine in aXis2000. The morphology and the texture of the biominerals were investigated with a field emission gun (FEG) ZEISS Ultra55 scanning electron microscopy (Zeiss, Marly-le-Roi, France) equipped with an X-ray energy-dispersive spectroscopy (XEDS) probe (XFlash 4010; Bruker). The samples were imaged in back-scattered electron mode at 15 kV (working distance of 7.5 mm) or in secondary electron mode at 3 kV (working distance of 3 mm). In addition, the samples were analyzed by scanning transmission electron microscopy (STEM) in high-angle annular dark field mode, by high-resolution TEM, and by XEDS, using a JEOL2100F FEG-TEM (JEOL, France) operating at 200 kV. Selected area electron diffraction (SAED) patterns were obtained on areas of interest and used to characterize amorphous and (nano)crystalline mineral phases.

### Electrochemical Characterizations

Electrochemical analyses were conducted in laboratory Swagelok-type cells. Solid phases collected from the experiments were either used as they were or crushed for 6 min in 25-ml jars with two 2-g steel balls and 50 mg of powder in a high-energy ball mill SPEX 8000. As deduced from the TGA results, the proportion of minerals (MnO_2_) was used to calculate the mass of active material (AM, corresponding to the mass of MnO_2_ only) in the electrode, which did not take into account the organic matter and water parts. The positive electrodes were prepared by hand-mixing (20 min in a mortar in air) the AM powder with 25 wt% of Super-P (SP) carbon, followed by 20 min of drying at 50°C. The cells were assembled in an argon-filled dried box. Then, 5–10 mg of AM-SP assemblage was separated from the negative electrode (lithium foil) by two glass fiber disks, the whole being soaked in LiPF_6_ (1 M) solution in ethylene carbonate/dimethylcarbonate mixture (1/1 w/w) (LP30, Merck). Galvanostatic cycling tests were conducted at room temperature in the 2–3.9 V potential window, with a rate cycling of C/20 (one electron exchanged in 20 h) using a MacPile controller (Claix, France). The power rate cycling was performed following the protocol by Doyle et al. (1994), under the same conditions but at different cycling rates (from 5 C to C/50), with a 1-h time separating the measurements at each rate. Specific capacities are reported in mAh per gram of AM.

## Results and Discussion

### Characterization of Biominerals

#### Uptake of Mn(II)

Whereas no precipitation could be observed by the naked eye under abiotic conditions (i.e., in the control without bacteria) in the timeframe of the experiment (2 weeks), Mn(II) oxidation was evidenced by the rapid tanning of the medium (after 10 h of incubation) in the presence of 2 × 10^8^ cell ml^–1^ *P*. *putida* MnB1 in 150 ml of biomineralization medium (Mn-Bio_150__ml_) due to the precipitation of Mn-bearing particle aggregates (Supplementary Figure SI1). The chemical analyses indicate that 1,740 μM out of the total added Mn(II) (1,800 μM over 2 weeks) was precipitated by the end of the experiment (Figure 1). Despite the relatively high total amount of Mn(II) added (1,800 μM) compared to previous studies (i.e., 10 μM–1 mM) (Okazaki et al., 1997; Nelson et al., 1999; Francis and Tebo, 2002; Villalobos et al., 2003; Parikh and Chorover, 2005; Templeton et al., 2005; Toner et al., 2005; Geszvain et al., 2013; Holguera et al., 2018), the overall yield of manganese precipitation stood between 95 and 99% after each addition of Mn(II) every day from the 2nd day to the 14th day (Figure 1). This is in the same range as the Mn(II) uptake quantified in Toner et al. (2005). Thus, the biomineralization of manganese by *P*. *putida* was highly efficient. In addition, contrary to previous studies performed over short time scales (typically 48–72 h) (Okazaki et al., 1997; Francis and Tebo, 2002; Villalobos et al., 2003; Toner et al., 2005), we observed that bacterial oxidation was active over longer periods (14 days).

#### Characterization of Biominerals Formed in Mn-Bio_150__ml_

**FIGURE 2:**
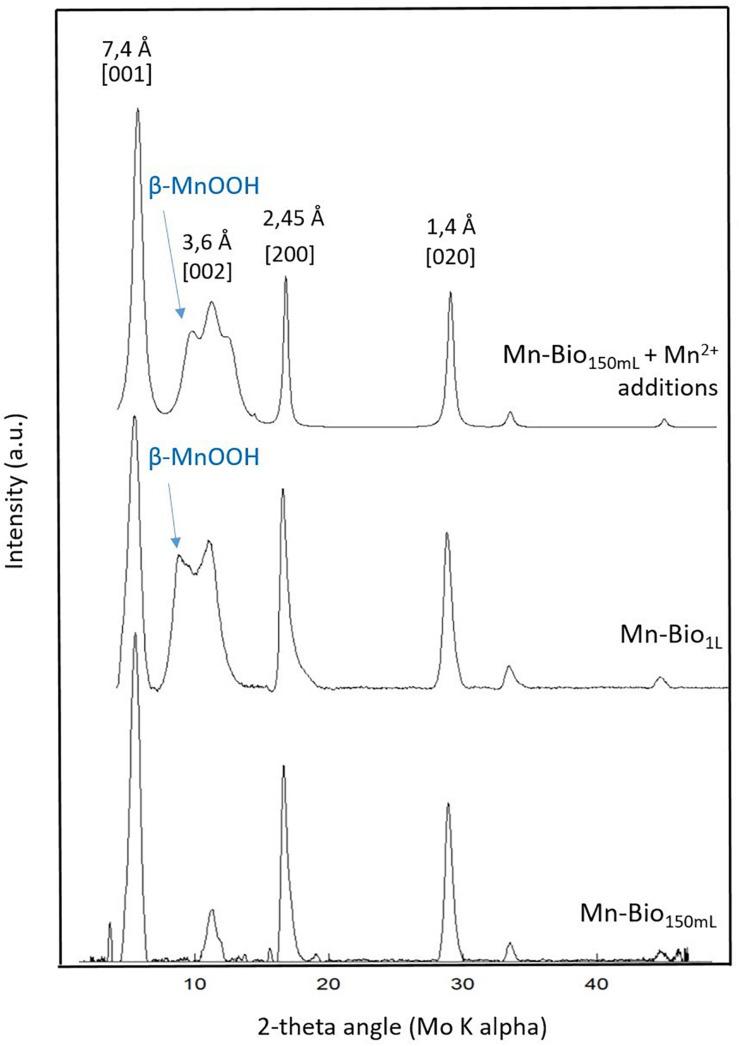
X-ray diffraction analyses of the biominerals obtained in Mn-Bio_1__l_, Mn-Bio_150__ml_, and Mn-Bio_150__ml_ after addition of Mn^2+^. Indexations correspond to birnessite. β-MnOOH is additionally observed in Mn-Bio_1__l_ and in Mn-Bio_150__ml_ + Mn^2+^ additions.

The XRD patterns of the biominerals obtained in Mn-Bio_150__ml_ (Figure 2) matched the reported structure of 7 Å-birnessite [JCPDS #00-018-0802 (Villalobos et al., 2003, 2006)] well. In accordance with our observations, most of the biogenic manganese minerals are reported to be very poorly crystallized (Tebo et al., 2004). The XRD patterns obtained here most closely resemble the poorly ordered hexagonal birnessite previously observed with *P*. *putida* (Villalobos et al., 2003, 2006) or with other Mn-oxidizer bacteria such as *Bacillus* SG-1 (Bargar et al., 2005; Webb et al., 2005). In contrast, we did not detect any feitknechtite or hausmannite as reported in other Mn biomineralization studies (Tebo et al., 2004; Bargar et al., 2005; Chen et al., 2017).

It is well-known that the interlayer space of such an open structure can host many species, especially metal cations. Despite the complex chemical composition of the culture medium, no extra-metals (e.g., Na) could be detected in the biomineral by XEDS (Supplementary Figure SI2), and no metal-mix oxide (e.g., Na–Mn–O-bearing phases) could be identified in the powder recovered after its annealing under air at 900°C.

**FIGURE 3:**
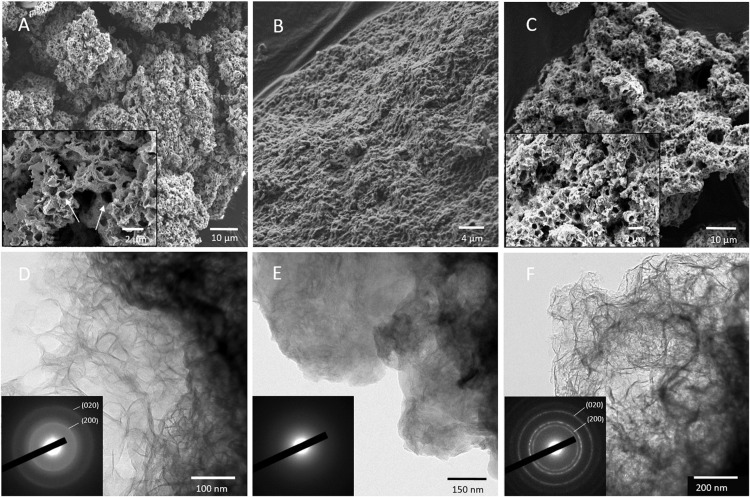
**(A–C)** Scanning electron microscopy and **(D–F)** transmission electron microscopy (TEM) analysis of the biominerals formed in **(A,D)** Mn-Bio_150__ml_, **(B,E)** Mn-Bio_1__l_, and **(C,F)** Mn-Bio_150__ml_ + Mn^2+^ additions. The inset in **(A)** shows that the multi-scale porosity (arrows) is obtained only in small volumes of the culture. In Mn-Bio_150__ml_ and Mn-Bio_150__ml_ + Mn^2+^ addition conditions, the electron diffraction in the TEM [inset in **(D,F)**] is consistent with a birnessite. In contrast, the mineral obtained in large volumes of culture has an amorphous electron diffraction pattern [inset in **(E)**].

**FIGURE 4:**
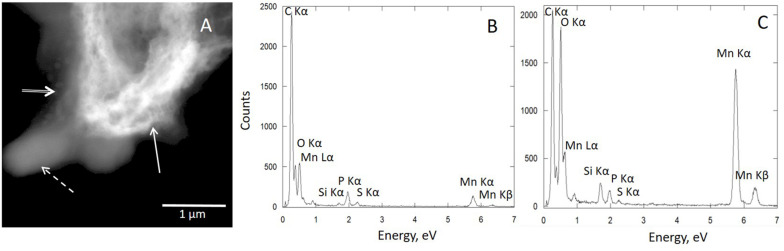
Scanning transmission electron microscopy (STEM)–X-ray energy-dispersive spectroscopy (XEDS) analysis of the bacteria–mineral assembly of Mn-Bio_150__ml_: **(A)** STEM image showing the bacteria (dotted arrow), polyphosphate granules (double arrow), and extracellular biomineral (solid arrow). **(B)** corresponding XEDS spectrum of bacteria and **(C)** corresponding XEDS spectrum of extracellular biomineral. **(B)** Particles composed of both Mn and P are closely associated with organic matter.

The Mn-Bio_150__ml_ biominerals consisted of micrometer-sized polydisperse aggregates of nanoparticles (SEM; Figure 3A and Supplementary Figure SI3). The agglomerates were highly porous, with pore sizes ranging from 100 nm to 1.2 μm (mean size: 500 ± 200 nm) (Figure 3A). The TEM images (Figure 3D) showed very thin, interlaced folded layers. The biominerals were extracellular but were co-localized with organic matter, most probably extracellular polymeric substances (EPS) which formed an organic network around the manganese oxides (Figure 4A). The SAED patterns (Figure 3D) confirmed a birnessite structure even though the small coherent crystalline size imparted a broadening of the diffraction rings. Using XEDS analyses combined with STEM observations, the particles composed of both Mn and P were identified in close proximity with organic matter or cells (Figure 4B), whereas phosphate-poor particles (birnessite) were rather observed at a distance from the cells (Figure 4C). A multitude of different textures and morphologies are reported for biogenic manganese oxides (Jiang et al., 2010; Mayanna et al., 2015; Chen et al., 2017), depending in particular on the stirring conditions of the culture (Cheney et al., 2008). The porous texture obtained in our study was never described for biogenic manganese oxides produced by the *Pseudomonas* strain.

The thermal analyses (TGA/DSC) and mass spectrometry (MS) data (Supplementary Figure SI4) showed three distinct phenomena taking place upon heating in air. The first one, up to 150°C, corresponded to the endothermic desorption of water (∼9% of the total mass of the material), probably loosely bound to the solid. The subsequent process was exothermic, with a massive release of water and CO_2_ (∼45%), matching the combustion of organic matter. Then, came another exothermic process, also releasing CO_2_ but much less water, probably clueing the combustion of carbon-rich organic residues (Mirvaux et al., 2016). Then, after a total weight loss of ∼67 wt%, transformations dwelt from ∼ 600°C with no further thermal signal. Thus, we can estimate the mineral part (MnO_2_) to account for ∼33 wt% of Mn-Bio_150__ml_. The material produced by biomineralization is therefore a composite material, composed of mineral as well as organic material. Organic matter, of which the cells have been inactivated by drying, could be considered as dead matter in the composite material. The XRD analyses indicated that the brown powder recovered after the thermal run was mainly composed of Mn_3_O_4_, with the presence of a small amount of Mn_3_(PO_4_)_2_. The presence of both organic matter and manganese phosphates in Mn-Bio_150__ml_ prompted us to further investigate its intimate organization and local composition.

**FIGURE 5:**
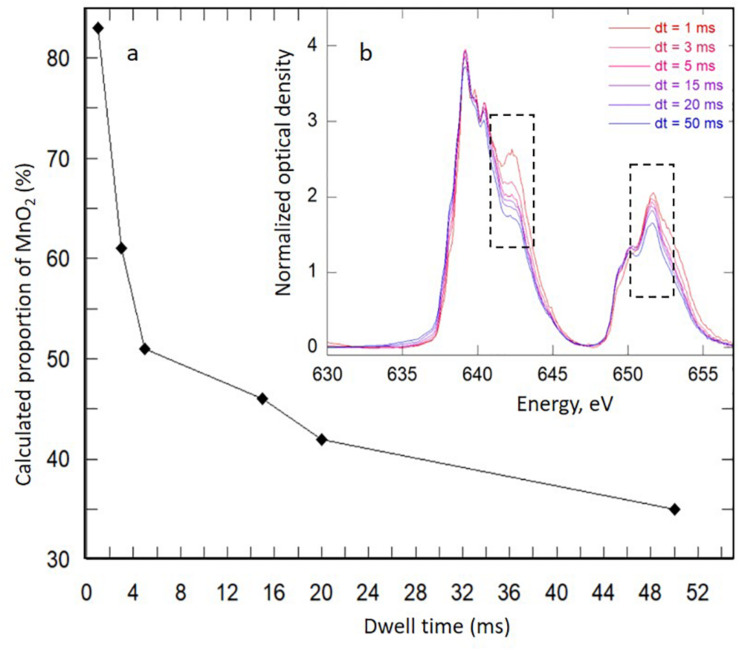
**(a)** Evolution of the proportion of reference birnessite in Mn-Bio_150__ml_ calculated from **(b)** Mn L_2,3_-edges near-edge X-ray absorption fine structure fitting of spectra recorded on a biomineral-rich area and analyzed at increasing dwell times. The dotted rectangles in **(b)** correspond to the parts of the spectra most strongly impacted by radiation damage.

Upon STXM analyses, the Mn phases were highly sensitive upon exposure to the X-ray beam (Figure 5). The Mn L_2,3_-edges spectra obtained at increasing dwell times on biomineral-rich areas in the Mn-Bio_150__ml_ sample were fitted with a combination of reference birnessite (H_0.5_MnO_2_, based on the redox state obtained from permanganometry) and HMn(II)PO_4_. Extended exposure to the beam induced modifications of the spectra both at the Mn-L_3_ and the Mn-L_2_ edges. For the typical dwell times used during analyses (1–3 ms per energy and image point), approximately 20% of the Mn(III)–Mn(IV) signal was lost, and this proportion increased at longer exposure times. Consequently, radiation damage led to Mn(III)–Mn(IV) reduction to Mn(II) at the local scale provided by STXM. The beam-induced Mn(IV)–Mn(III) reduction may have been enhanced by the presence of associated organic matter. As described by Toner et al. (2005), the free-radical species produced upon exposure of organic matter to X-rays could cause the observed photoreduction.

**FIGURE 6:**
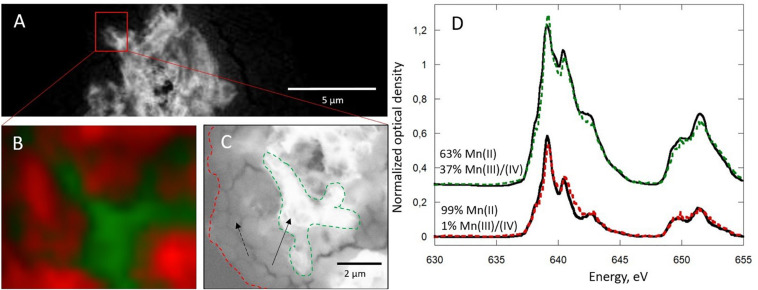
Scanning transmission X-ray microscopy analysis at the Mn L_2,3_-edges of Mn-Bio_150__ml_ after 5 days. **(A)** Mn map (640–630 eV). **(B)** Color-coded composite map of the area squared in **(A)** showing two components, in which the Mn L_2,3_-edges near-edge X-ray absorption fine structure spectra are given in **(D)**, with corresponding results of linear combination fitting. **(C)** The same area has been analyzed by scanning electron microscopy, allowing to distinguish the two components. Red, Mn associated with organic matter (dashed arrow); green, Mn oxide (solid arrow).

The presence of areas almost exclusively composed of Mn(II), even at short dwell times, suggests the presence of Mn(II) phases in small proportions in the composite material (Figure 6). Combined STXM and SEM analyses confirm the co-localization of organic matter and Mn(II) (Figure 6C). Mn(II) may be adsorbed at the surface of organic matter or precipitated as manganese phosphate (Figure 6B). An increase of phosphate and protein concentrations in cells and the extracellular environment upon Mn(II) oxidation could explain the formation of manganese phosphate close to organic matter (Parikh and Chorover, 2005). In addition, the hydrolysis of polyphosphate granules, such as those observed in some cells (Figure 4A), could provide a source of inorganic phosphate that may react with Mn(II) sorbed at the cell surface, leading to manganese phosphate precipitation near the cells.

#### Effect of O_2_ Supply and Excess Dissolved Mn(II)

It has been suggested that the oxidation of Mn(II) may result from a combination of biological (enzymatic catalysis) and abiotic (surface-driven catalysis) processes (Diem and Stumm, 1984; Tebo et al., 2004; Bargar et al., 2005; Webb et al., 2005), both being also dependent on the amount of available Mn(II) in the medium (Bargar et al., 2005; Zhao et al., 2016) and on oxygen supply, i.e., transport of atmospheric oxygen toward the Mn(II) oxidizing sites. To some extent, the amount of available Mn(II) in the medium or oxygen supply may also alter the structure, texture, or morphology of the precipitates (Cheney et al., 2008). It is worth noting that dissolved Mn(II) concentration can orientate precipitation toward different polymorphs (γ-MnO_2_ δ-MnO_2_) (Tu et al., 1994). In this framework, we explored the impact of both oxygen supply and Mn(II) concentration on the mineralogy of Mn-bearing solids formed in our system.

First, we modified the O_2_ supply to the biomineralization medium using a larger volume of biomineralization medium (1 L instead of 150 ml), all other parameters, including the stirring method, being kept alike. In this configuration, the O_2_ supply to the Mn(II) oxidizing sites was lower because of the smaller ratio between (i) the surface area of air/medium contact and (ii) the volume of the medium. The resulting Mn-Bio_1__l_ precipitate consists of compact aggregates of small particles devoid of any apparent porosity (Figure 3B), and XRD analyses indicated a mixture of birnessite and feitknechtite (β-Mn^III^OOH) phases (Luo et al., 2000; Figure 2). Mn-Bio_1__l_ and Mn-Bio_150__ml_ are thus texturally, chemically, and structurally very different, whereas only the volume of culture changed. The presence of feitknechtite suggests that the lower O_2_ supply (1 L experiment) decreased the ratio of rates of Mn(II) to Mn(IV) oxidation vs. Mn(II)–Mn(IV) interaction, leading to Mn(III). Moreover, the surface of the biomineralized birnessite may promote the abiotic (chemical) oxidation of dissolved Mn(II) [preferably present in Mn-Bio_1__l_ because of higher concentrations of Mn(II) in the medium] into Mn(III) as already reported (Diem and Stumm, 1984).

**FIGURE 7:**
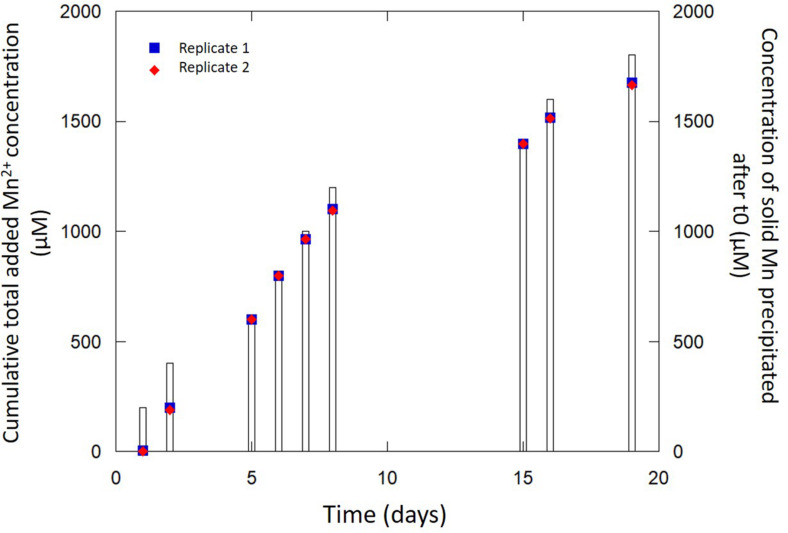
Evolution of Mn incorporation upon abiotic reaction of Mn-Bio_150__ml_ biominerals with Mn^2+^ (blue squares and red diamonds). The cumulative total added Mn^2+^ concentration (bars) increases with time following daily additions of Mn^2+^.

Secondly, in order to further disentangle the abiotic from the bio-driven processes, we monitored the fate of dissolved Mn(II) added daily to a bacteria-free biomineralization medium (150 ml) initially containing only biomineralized birnessite particles (30 mg of dried Mn-Bio_150__ml_). Whereas no reaction was spotted upon the addition of Mn(II) in a medium free of any Mn-bearing precipitate, the presence of birnessite in the suspension clearly promoted the consumption of Mn(II) to form solids (Figure 7). After a latent phase (day #1), we observed the formation of additional Mn solids from the added Mn(II). From day #5 to day #18, the rate of Mn(II) consumption increased. After 18 days, the proportion of added Mn(II) that underwent precipitation reached 95%. The efficiency of this process might be related to (i) the high surface of contact and open porosity of Mn-Bio_150__ml_ (Figure 3), (ii) the presence of organic matter associated with the mineral in Mn-Bio_150__ml_ that could act itself as a catalyst of Mn(II) oxidation despite hindering a part of the mineral active surface, and (iii) the ability of organic matter to adsorb Mn(II) ions (Parikh and Chorover, 2005). A component resulting from this chemical process was clearly identified as β-Mn(III)OOH by XRD (Figure 2), hence the same product as that formed in the previous 1 L-experiment. The SEM observations of Mn-Bio_150__ml_ + Mn(II) additions showed a modification of the surface/texture of Mn-Bio_150__ml_, making pores less visible (Figure 3C), whereas TEM observations did not show significant differences with the powder before Mn(II) additions (Figure 3F).

These experiments highlight a competition between bacterial (enzymatic) and chemical (abiotic) oxidation of Mn(II). The presence of dissolved Mn(II) could lead to the partial dissolution of birnessite and the precipitation of a new Mn(III)-bearing mineral (Lopano et al., 2007). This observation suggests that Mn oxides are extremely labile and quickly respond to thermodynamic driving forces (Webb et al., 2005). A rapid conversion of feitknechtite into birnessite was observed in another study when the Mn(II) concentration in the experiments was reduced below thermodynamic stability. On the contrary, excess Mn(II) promoted the conversion of birnessite to feitknechtite (β-Mn^*III*^OOH) (Bargar et al., 2005). The presence of structural Mn(III) could be explained by Mn(IV) reduction by excess Mn(II) in solution (Silvester et al., 1997). In order to favor the precipitation of birnessite containing a majority of Mn(IV) and hinder the formation of feitknechtite, the bio-enzymatic process has to be predominant. This implies optimal culture conditions, in particular a slow addition of Mn(II) together with a high O_2_ supply. Otherwise, the abiotic reaction leads to the important formation of Mn(III), resulting in the precipitation of β-Mn^III^OOH together with birnessite.

### Electrochemical Performance in Li Half-Cells

MnO_2_ has been used and investigated as an electrode material for Li batteries in multiple studies (Lee and Goodenough, 1999; Jeong and Manthiram, 2002; Toupin et al., 2002; Kim and Popov, 2003; Brousse et al., 2006; Alfaruqi et al., 2015; Qiu et al., 2018). It provides capacities close to the theoretical capacity (308 mAh g^–1^), depending on the size and the morphology of the particles and the cycling conditions. Bach et al. (1991) synthetized hydrated nano-particles of birnessite *via* a sol–gel process at 60°C. The working electrode consisted of either a stainless steel or a gold grid with a geometric area of 0.5 cm^2^, on which the birnessite mixed with graphite (20 or 90 wt%) was pressed. In the potential range of 2–4.5 V vs. Li^+^/Li° at room temperature, this material exhibited a capacity of 250 mAh g^–1^ for a rate of 10 μA cm^–2^. The layered MnO_2_ produced by Ma et al. (2004) consisted of abundant quasi-1D nanomaterials made by hydrothermal synthesis, exhibiting a capacity of 375 mAh g^–1^ for the first cycle but with a considerable capacity loss during the second cycle. This first-cycle high capacity can be explained by the fact that the redox couple Mn^4+^/Mn^3+^ was not the only one involved in this electrochemical reaction. The cells were cycled with 37% of conductive carbon in the voltage range 1.0–4.8 V vs. Li^+^/Li° at a constant current density of 0.23 mA cm^–2^ (mass capacity of 2.0 mA g^–1^) at 25°C. Finally, Thapa et al. (2014) synthetized a mesoporous birnessite composed of nanometer-scale particles (50–100 nm) with the oxidant KMnO_4_ at 4°C. This mixture was pressed onto a stainless steel mesh. The average thickness of the cathode electrode was around 18–20 μm (10 mg per active mass of cathode electrode at 2 cm^2^), providing a capacity of 305 mAh g^–1^ close to the theoretical value at a C/30 rate in the potential range of 1.5–4 V vs. Li^+^/Li°. However, the capacity of this material continuously decreased with the number of cycles to reach about 150 mAh g^–1^ at the 20th cycle and 100 mAh g^–1^ at the 50th cycle.

**FIGURE 8:**
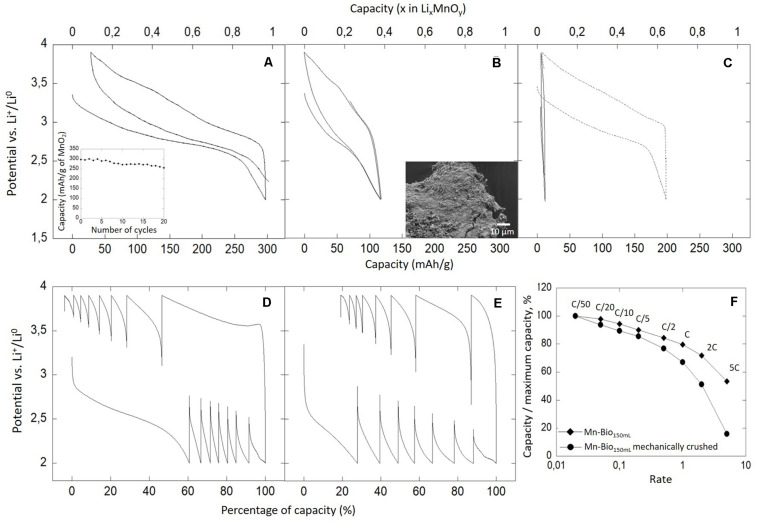
Electrochemical analyses vs. Li^+^/Li° of **(A,D)** Mn-Bio_150__ml_, **(B,E)** mechanically crushed Mn-Bio_150__ml_, and **(C)** Mn-Bio_150__ml_ + Mn^2+^ additions (dotted curve) and Mn-Bio_1__l_ (solid curve). **(A,B)** Galvanostatic cycling curves were obtained with 25 wt% SP-C at C/20 (1 Li in 20 h) and the corresponding capacity retentions in charge are given. **(D,E)** Corresponding power rates. **(F)** Power rate in charge of Mn-Bio_150__ml_ and of the mechanically crushed Mn-Bio_150__ml_.

Here we investigated the electrochemical activity of the biominerals formed by *P*. *putida* vs. Li^+^/Li°. The galvanostatic curves obtained for a Mn-Bio_150__ml_–SP mixture revealed a reversible process at an average potential of 3.2 V vs. Li^+^/Li° (Figure 8A and Supplementary Figure SI5), in agreement with the Mn^4+^/Mn^3+^ redox couple activity (Manthiram and Kim, 1998). From TGA/MS analyses, we estimated the mass of MnO_2_ to represent 33 wt% of the composite material. As a consequence, we calculated that this material provided a reversible capacity of 297 mAh g^–1^ (Δ*x* = 0.97 Li per MnO_2_), i.e., 97% of the theoretical capacity of MnO_2_ at a C/20 rate (Figure 8A). This corresponds to a surface current density of 30 μA cm^–2^ and a mass capacity of 30 mA g^–1^. This capacity was very close to the theoretical one and confirmed that Mn-Bio_150__ml_ was mainly composed of Mn(IV). Biomineralized MnO_2_ provided a capacity in the high range of those reported in the literature in the same potential range (2–4 V, field of the Mn^4+^/Mn^3+^ redox couple activity) (Bach et al., 1991; Ma et al., 2004; Thapa et al., 2014). In addition, the biominerals showed a good cycling stability, with only a 15% capacity loss over the 20 first cycles (Figure 8A, insert). In contrast, abiotic MnO_2_ was reported to lose 50 mAh g^–1^ (Thapa et al., 2014) or 85 mAh g^–1^ (Ma et al., 2004) between the first and the second cycles.

As reported in the literature, the capacity of MnO_2_ could be altered after a certain number of cycles due to MnO_2_ dissolution and reduced specific surface (Tompsett et al., 2013) rather than due to a structural distortion of MnO_2_ (Chae et al., 2013; Mathew et al., 2014). In our present study, we observed a degradation of the texture of biogenic manganese oxides after 100 cycles that could be related to organic matter degradation and could account for the long-term decrease of capacity (Supplementary Figure SI6). The characterization and the evolution of organic matter upon cycling would deserve a future dedicated study.

We also evaluated the capacity of the biominerals as a function of cycling rate. The power rate plots (Figures 8D,F) showed that Mn-Bio_150__ml_ maintained its redox activity at high rates with, for instance, 80% of the maximum capacity still available at 1C and 60% at 5C. This property could be due to the porosity of the biomineralized birnessite induced by the EPS network, which could enhance electrolyte circulation and thereby ionic transfer, as shown with biomineralized iron oxides (Miot et al., 2014) and iron phosphates (Mirvaux et al., 2016).

In order to confirm the impact of biomineral texture on electrochemical performances, we ball-milled Mn-Bio_150__ml_. As expected, the SEM observations revealed a loss of texture (Figure 8B). We compared the electroactivity of Mn-Bio_150__ml_–SP before and after ball-milling. After ball-milling, the galvanostatic curves indicated an average specific capacity of 117 mAh g^–1^ for the first cycle at C/20 (Figure 8B), much lower than for non-milled Mn-Bio_150__ml_–SP. Similarly, the rate capability of milled Mn-Bio_150__ml_–SP was found lower than that of pristine Mn-Bio_150__ml_–SP. Indeed with, for instance, 60% of the maximum capacity available at 1C and 10% at 5C in the first charge, there was a 50% loss of specific capacity compared to the initial biomineral (Figures 8E,F).

As a conclusion, the texture of the electrode material plays a key role in electrochemical performances. The role of the texture has already been highlighted and could ensure electrolyte penetration and the preservation of particle interconnectivity and provide a conducting network (Miot et al., 2014). As observed for heated FP-bacteriomorphs (Mirvaux et al., 2016), the microbially inherited texture allows to bypass energetic ball-milling with conducting carbon.

In contrast, the Mn-Bio_1__l_–SP mixture did not exhibit any electrochemical activity (Figure 8C). This can be explained, on the one hand, by the presence of β-Mn(III)OOH which is a “dead weight” and ionically insulating phase and, on the other hand, by the very limited porosity of this sample (Figure 3B) which prevents electrolyte percolation, thus negatively impacting the effective ionic conductivity. This last hypothesis could be further fed by studying the material resulting from Mn(II) addition experiments, which contained β-Mn^III^OOH but still had some porosity. Indeed Mn-Bio_150__ml_ + Mn(II)–SP exhibited a first discharge capacity of 197 mAh g^–1^ (Δ*x* = 0.64 Li per MnO_2_) (Figure 8C), i.e., only 30% less than that of Mn-Bio_150__ml_. The presence of a β-Mn(III)OOH phase thus negatively impacted the electrochemical performance of the biomineral, but due to its biologically inherited porous texture, the material retained some electroactivity.

## Conclusion

*P*. *putida* MnB1 promoted the oxidation of Mn(II) in aqueous medium, leading to the precipitation of 95% of soluble manganese added. This oxidation process was still efficient even after more than 14 days, and a cumulated concentration of 1.8 mM Mn(II) was provided. The XRD and TEM analyses revealed the formation of birnessite. The electrochemical capacity of this mineral, close to the theoretical one, confirmed that Mn-Bio_150__ml_ consisted mainly of Mn(IV). Minor proportions of Mn(II) either adsorbed or precipitated in the form of Mn(II)-phosphate were also observed in this material, in association with organic matter. As shown by the thermogravimetric analyses, the biomineral was a composite material hosting ≈67% organic matter. SEM and TEM revealed that the extracellular biomineralized birnessite consisted of highly porous aggregates of MnO_2_ nanoparticles. These biominerals (Mn-Bio_150__ml_) exhibited a good capacity and a high power rate in semi-battery configurations vs. Li^+^/Li°. Such an enhanced electroactivity relied on the specific texture of the biominerals inherited from biomineralization. Indeed the mechanically milled biominerals exhibited a degraded electroactivity. It would be interesting to explore the degradation of organic matter upon cycling and to measure its impact on capacity.

In addition, we evidenced a competition between enzymatic oxidation of Mn(II) into MnO_2_ and abiotic reactions between soluble Mn(II) and the already-formed MnO_2_. Above a critical rate of this latter reaction, the formation of Mn(III)-bearing phases was observed, with an adverse impact on the electrochemical performance of the material despite a similar porous texture. Consistently, we observed that biomineralization under less oxygenated conditions (Mn-Bio_1__l_) resulted in a loss of porosity and enhanced formation of Mn(III) phases annihilating any electroactivity.

Thus, the specific texture inherited from microbial biomineralization, as well as the control over the birnessite-to-Mn(III)-phases ratio, through the control of environmental parameters such as oxygenation level and rates of Mn(II) supply, appears to be key in providing Mn oxides with a good and stable electrochemical performance. Controlling the conditions of biomineralization and exploring the diversity of Mn-oxidizing microorganisms and their various Mn oxidation pathways and culture conditions open possibilities to optimize biosynthesis and extend applications to other Mn^4+^/Mn^3+^-bearing electroactive materials.

## Data Availability Statement

All datasets generated for this study are included in the article/Supplementary Material.

## Author Contributions

JM, FG, and NR conceived and planned the experiments. LG and NR performed the experiments. FS-P helped with microbiology experiments on IMPMC Biology Plateform (GEMME). JM, FG, NR, and LG performed the sample analyses. NR, FG, DL, JM, and LG contributed to the interpretation of the results. LG wrote the manuscript with critical feedbacks from all authors. All authors contributed to the article and approved the submitted version.

## Conflict of Interest

The authors declare that the research was conducted in the absence of any commercial or financial relationships that could be construed as a potential conflict of interest.
