# Supplementary material for: Microbially Induced Mineralization of Layered Mn Oxides Electroactive in Li Batteries
Source: Front Microbiol. 2020 Sep 10;11:2031. doi: 10.3389/fmicb.2020.02031 (PMC7511517; doi:10.3389/fmicb.2020.02031)
Supplement: Supplementary file 1 [file Data_Sheet_1.docx]

Supplementary Material

# Supplementary Figures


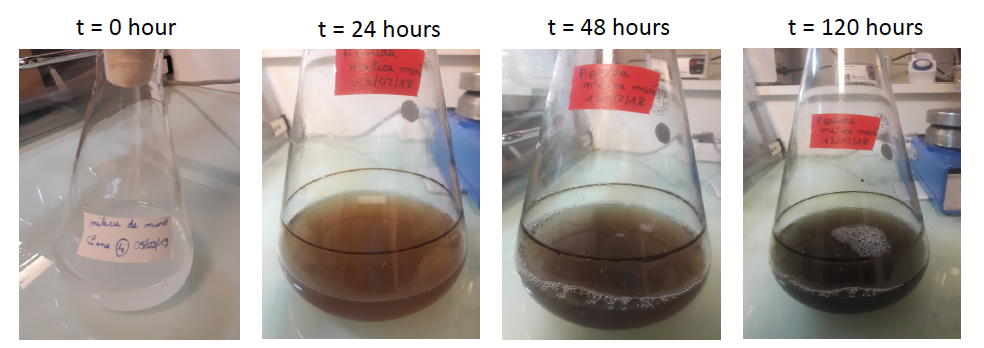


**Supplementary Figure 1.** Evolution of 150 mL biomineralization medium inoculated with *Pseudomonas putida* (Mn-Bio_150mL_) following daily additions of Mn^2+^. Medium darkening, that starts 10 h after cell inoculation, corresponds to the formation of Mn oxides.

**
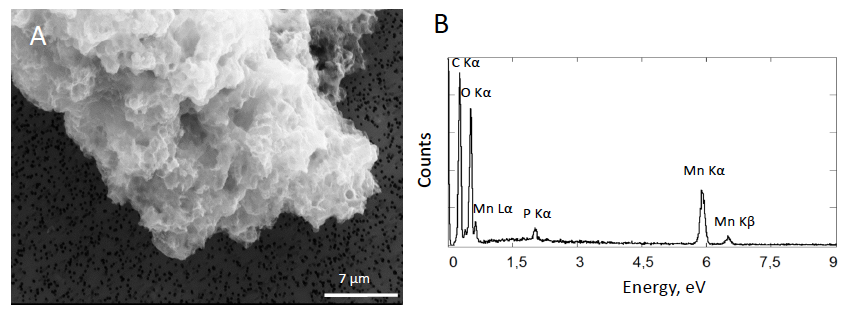
**

**Supplementary Figure 2.** SEM-XEDS analysis of Mn-Bio_150mL_ obtained after 14 days of incubation in the mineralization medium: (A) SEM image and (B) XEDS spectrum of the biomineral.


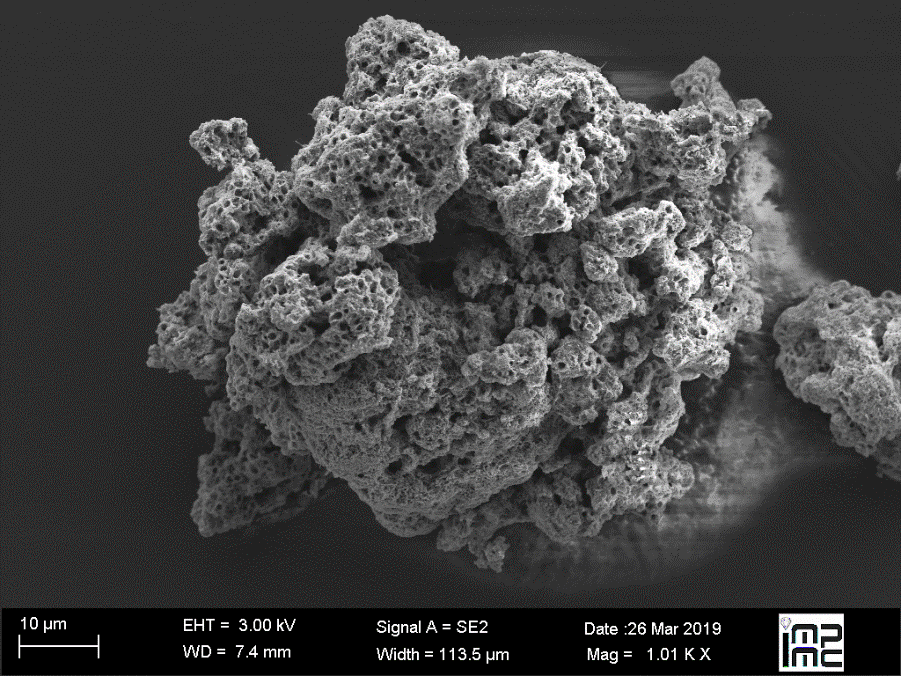

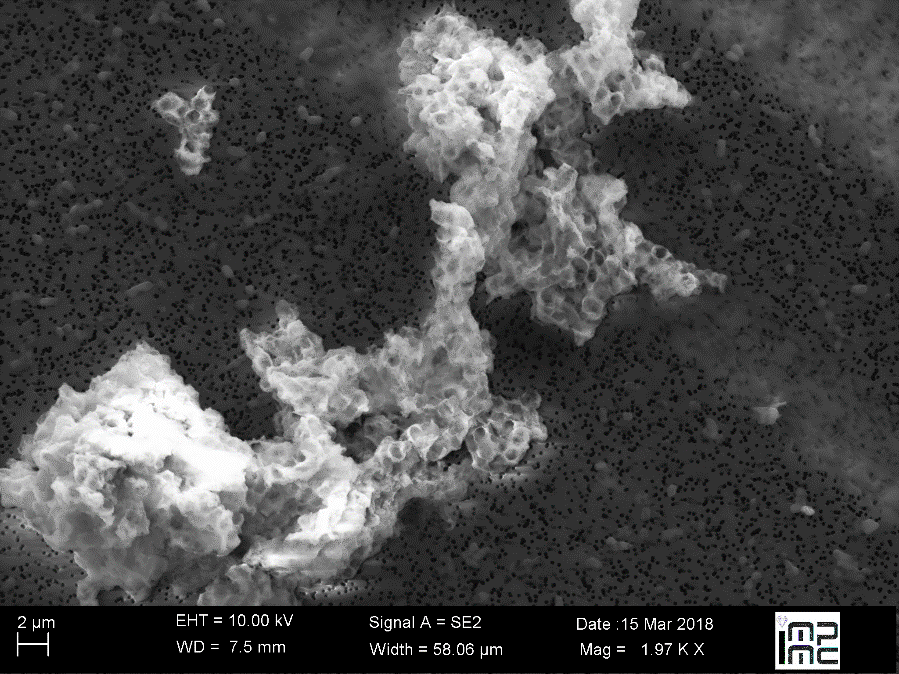

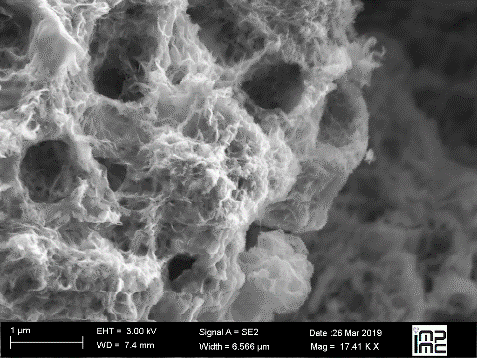


4 µm

10 µm

1 µm

A

B

**Supplementary Figure 3.** SEM observations of independent Mn-Bio_150mL_ experiments.


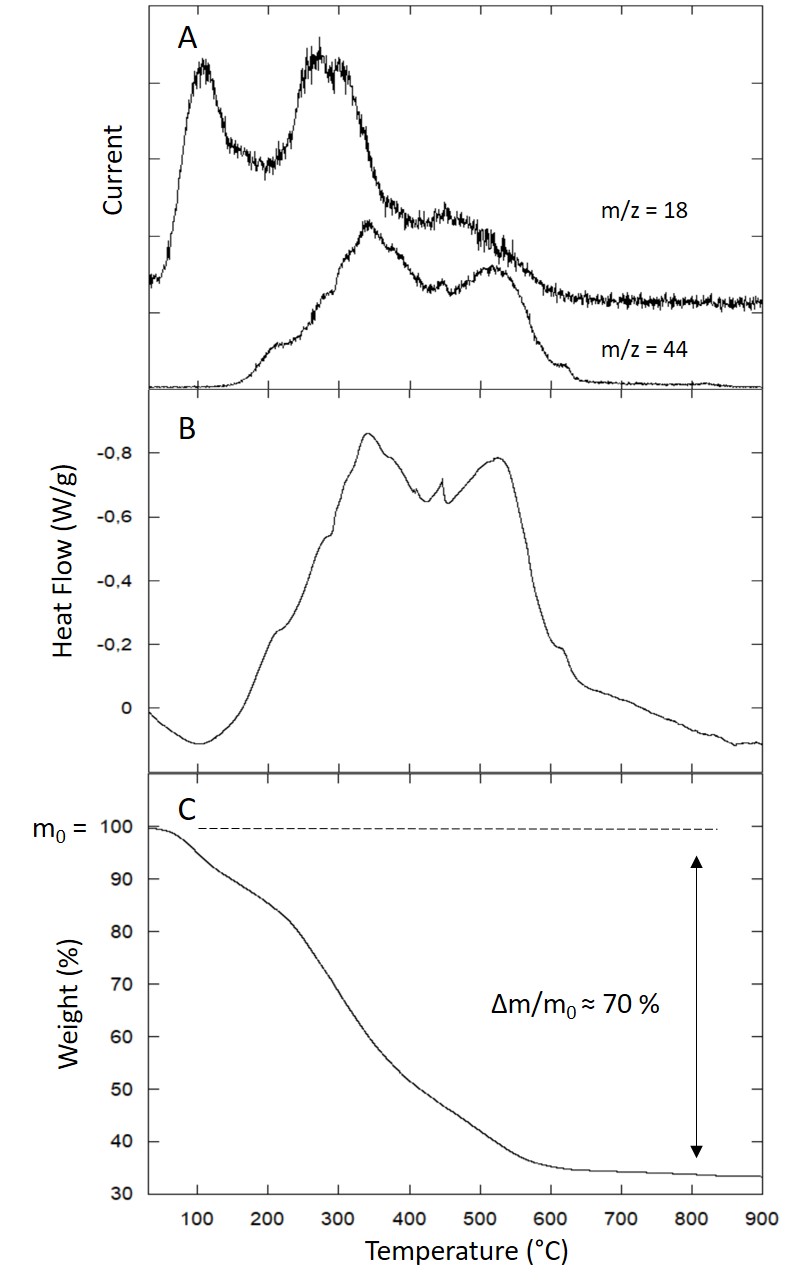


**Supplementary Figure 4.** (A) Mass Spectrometer (MS) signals recorded on the gas released during coupled: (B) Differential Scanning Calorimetry (DSC) and (C) Thermogravimetric analyses (TGA) for biominerals from Mn-Bio_150mL_. Only m/z = 44 (CO_2_) and m/z = 18 (H_2_O) traces are plotted.

**Supplementary Figure 5.** Galvanostatic cycling curves of independent Mn-Bio_150mL_ experiments vs Li^+^/Li° (A) : 25 wt% SP-C at C/10 (1 Li in 10 h) and (B) : 25 wt% SP-C at C/50 (1 Li in 50 h).

Potential vs. Li^+^/Li^0^

Capacity (x in Li_x_MnO_y_)


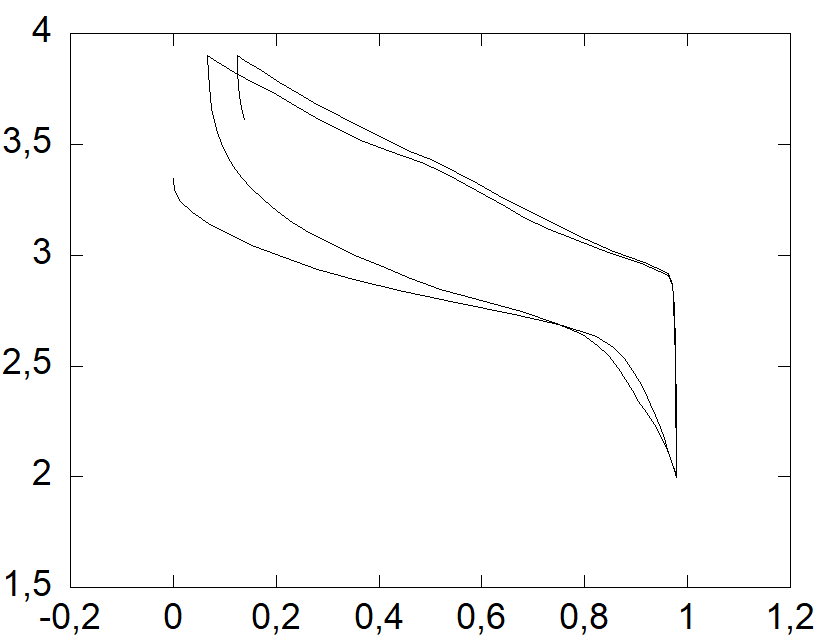

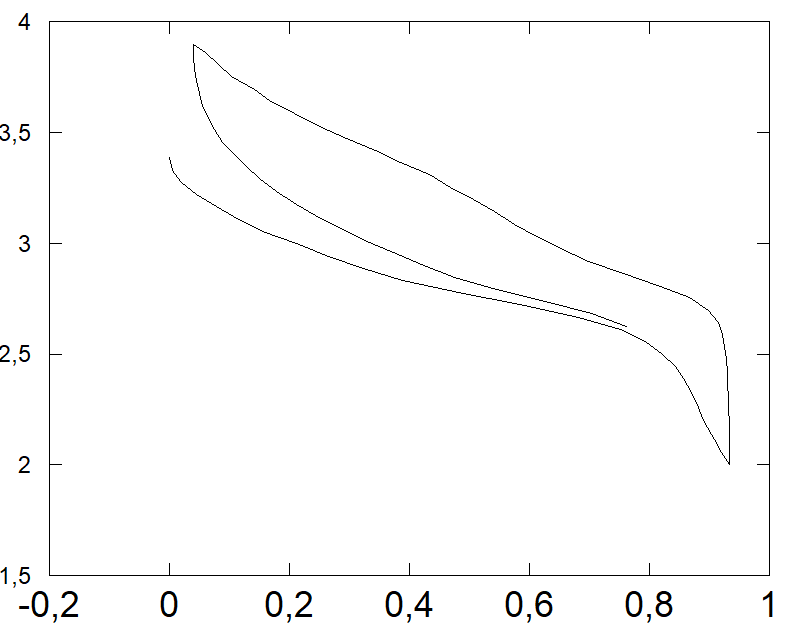


A

B


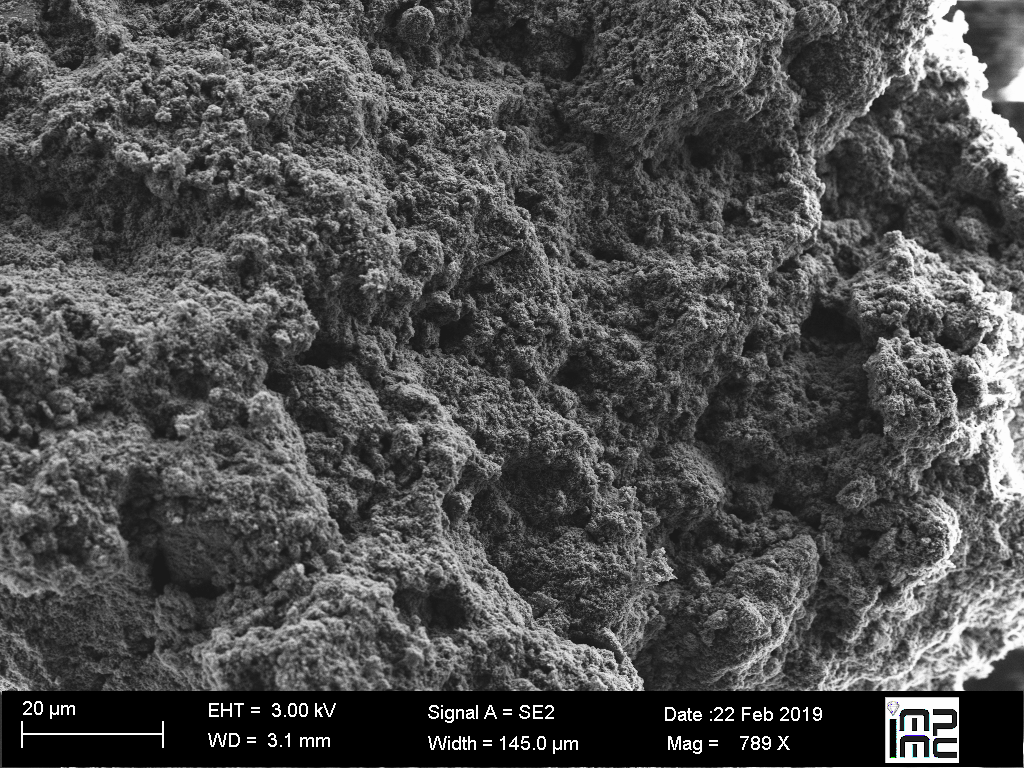


10 µm

**Supplementary Figure 6.** SEM observations of Mn-Bio_150mL_ after 100 cycles in battery.
